# Case of Malformation in a Child

**Published:** 1851-07

**Authors:** M. Emanuel

**Affiliations:** Marcus Hook, Pennsylvania; Del. Co.


*Case of Malformation in a Child.* By M. EMANUEL, M. D. Marcus Hook, Pennsylvania.

I was called to attend Mrs. H. in labor with her fifth child, and in about an hour she was delivered. In dividing the cord, I observed some malformation about the umbilicus, and as there was considerable flooding, I handed the child over to the nurse, and said nothing until after the expulsion of the placenta, to the upper end of which was attached a sac, containing a pint of coagulated blood. As soon as the mother was relieved, I examined the child, which had the following singular malformation: at each side of the umbilical opening, two small glands projected half an inch externally, resembling the testes, and very vascular. There was no penis, but a well formed scrotum, without testes, and no orifice at the anus. The right leg, from the knee joint to the ankle, was half the length of the other, and in place of a foot, there was a continuation of cartilaginous substance about four inches in length, resembling a finger, with an extensor and flexor attached to the end, moveable at will. I made an incision in the perineum, and introduced a female catheter without any difficulty, three inches up the rectum, but no discharge came away. Before leaving, I left directions with the nurse to notice if the child had any evacuations, and where they escaped. In all other respects it was a fine healthy looking child. I thought I would not say any thing to the mother at the time; but as I was leaving the room, she inquired if the child was marked with the figure of horse, as she said that a little before she found herself in the family way, she had a fright; that one of her children had fallen between the horse and cart, and she fancied the horse had kicked him and forced his entrails out. This impression was strongly on her mind during the whole period of her pregnancy, although the boy was very little hurt. On visiting her the next day, the nurse said the child took some food, was very lively, and that it had passed both urine and feces, but that it all escaped at the navel. On making further examination, I noticed something of the shape and size of a French bean between the two small glands before mentioned, with an orifice in the centre of the flat surface from which the urine escaped. On the second and third day the child took the breast, the evacuations continuing as before, although the passage into the rectum was perfectly free. On the night of the third day the child was taken sick, and died on the fourth day after its birth. I visited her every day, though several miles from my residence, with the intention of obtaining the child after its death, for the purpose of making a preparation. Unfortunately, on that day I was so much engaged that I could not get there, and they buried it immediately, I know not where, to prevent my having it. You are aware how difficult it is for country practitioners to get a post mortem examination, or obtain parts for preparation.

*Marcus Hook, Del. Co. June,* 1851.

